# Occurrence of Regulated and Emerging Iodinated DBPs in the Shanghai Drinking Water

**DOI:** 10.1371/journal.pone.0059677

**Published:** 2013-03-26

**Authors:** Xiao Wei, Xin Chen, Xia Wang, Weiwei Zheng, Dong Zhang, Dajun Tian, Songhui Jiang, Choon Nam Ong, Gengsheng He, Weidong Qu

**Affiliations:** 1 Key Laboratory of the Public Health Safety, Ministry of Education, Department of Environmental Health, School of Public Health, Fudan University, Shanghai, China; 2 Department of Occupational and Environmental Health, School of Public Health, Guangxi Medical University, Nanning, Guangxi, China; 3 National Engineering Research Center of Urban Water Resource (south), Shanghai, China; 4 NUS Environmental Research Institute, National University of Singapore, Singapore, Singapore; 5 Key Laboratory of the Public Health Safety, Ministry of Education, Department of Nutrition and Food Hygiene, School of Public Health, Fudan University, Shanghai, China; National University of Singapore, Singapore

## Abstract

Drinking water chlorination plays a pivotal role in preventing pathogen contamination against water-borne disease. However, chemical disinfection leads to the formation of halogenated disinfection by products (DBPs). Many DBPs are highly toxic and are of health concern. In this study, we conducted a comprehensive measurements of DBPs, including iodoacetic acid (IAA), iodoform (IF), nine haloacetic acids and four trihalomethanes in drinking waters from 13 water plants in Shanghai, China. The results suggested that IAA and IF were found in all the water treatment plants, with maximum levels of 1.66 µg/L and 1.25 µg/L for IAA and IF, respectively. Owing to deterioration of water quality, the Huangpu River has higher IAA and IF than the Yangtze River. Our results also demonstrated that low pH, high natural organic matter, ammonia nitrogen, and iodide in source waters increased IAA and IF formation. Compared to chlorine, chloramines resulted in higher concentration of iodinated DBP, but reduced the levels of trihalomethanes. This is the first study to reveal the widespread occurrence of IAA and IF in drinking water in China. The data provide a better understanding on the formation of iodinated disinfection byproducts and the findings should be useful for treatment process improvement and disinfection byproducts controls.

## Introduction

Drinking water chlorination plays an important role in preventing pathogen contamination against water-borne disease. However, chemical disinfection leads to the formation of halogenated disinfection by products (DBPs) due to reaction with natural organic matter (NOM), and result in potential health concerns [1]. Epidemiologic investigations have demonstrated the associations between exposure to DBPs in drinking water with cancers (bladder [2], colon [3] and rectum [4]), adverse birth outcomes [5]–[11] and birth defects [12], [13]. Toxicological assessments have also revealed that several DBPs (e.g.,3-chloro-4-(dichloromethyl)-5-hydroxyl-2(5H) -furanone (MX) and formaldehyde) are potential carcinogens [1]. Up to now, about 600–700 DBPs have been identified in drinking water [1], [14], and only a few of DBPs have been evaluated for adverse effects. Hence, only handful of regulations and guidelines have been established for DBPs; they include four trihalomethanes (THMs) (chloroform, bromoform, bromodichloromethane, and chlorodibromomethane) and five haloacetic acids (HAAs) (monochloroacetic acid, dichloroacetic acid, trichloroacetic acid, monobromoacetic acid and dibromoacetic acid) [15]–[17]. More importantly, some DBPs, especially newly emerging DBPs do not have maximum acceptable values and are not required for routine surveillance.

Iodinated DBPs (I-DBPs) is a class of emerging DBPs identified in drinking water [1]. The main I-DBPs include iodo-acids and iodo-trihalomethanes. Although they have been found in finished drinking water at ng/L to low-µg/L levels [18], recent studies have demonstrated that iodinated DBPs were more cytotoxic and genotoxic than their brominated and chlorinated analogues [18]–[24]. Among I-DBPs, iodoacetic acid (IAA) and iodoform (IF) have attracted much attention because IAA caused higher cytotoxicity and genotoxicity in mammalian cells than MX [25], one of the most mutagenic compounds in drinking water. IF, besides strong odor, is the most cytotoxic among iodo-trihalomethanes [20], and is known to be more toxic than the regulated THM_4_. Both IAA and IF could be found in drinking water with chlorine, chloramines, and ozone as disinfectant over the world. Earlier study has demonstrated that the water source of coastal regions often suffer from salt water intrusion and led to increase the formation of I-DBPs [18].

Shanghai is a coastal city in east China. The Huangpu and Yangtze Rivers are the two main water sources for Shanghai, which provide 76.3% and 23.7% of raw water supply, respectively. Because water sources are located in estuarine region, each year, from November to April, salt water intrusion accompany with tidal effects and bring along halogenate compounds to various water intake sources. In addition, high ammonia concentration and serious organic matter pollution caused high chemical oxygen demand, and this has become the major environmental and water quality challenge for both the Huangpu River [26] and Yangtze Rivers [27]–[29]. To reduce formation of DBPs [30], most water plants in Shanghai used free chlorine followed by combined chlorine with chloramines to disinfect drinking water. Therefore, salt water intrusion and organic pollution become critical factors affecting the formation of the DBPs, especially I-DBPs. Earlier studies have been focused on regulated DBPs, few investigations concerned the occurrence of more toxic I-DBPs in drinking water of Shanghai, although recent evidence indicated that I-DBPs are easily formed when waters are high in iodide and treated with chloramines [18], [19].

Thus, the objective of the present study was to carry out a comprehensive study on the formation of IAA, IF, THM_4_ and HAA_9_ in drinking water of 13 water plants of Shanghai that are fed by the Huangpu or Yangtze Rivers. The total daily water supply of 13 water plants is close to 1.6 billion gallons, which covered 80% municipal water consumption of Shanghai and provides water for 14 million residents. Subsequently, the impact of I-DBPs formation was also investigated. Our results demonstrated that DBPs varied markedly depending on their water sources, seasons and treatment processes. The findings suggest that the Huangpu River has higher IAA and IF than the Yangtze River. Pre-chloramines increased IAA and IF formation, compared with chlorine and ozone. To our best knowledge, this is the first systematic study undertaken within China. The data obtained will be valuable to regulators and water suppliers for iodinated DBP detection and control.

## Materials and Methods

### Ethics Statement

This study belongs to a non-profit project supported by Chinese Ministry of Science and Technology. All necessary permits were obtained for the described field studies and approved by Shanghai Municipal Water Affairs Bureau (2008ZX07421-004). The location is not privately-owned or protected in any way and the field studies did not involve endangered or protected species.

### Drinking Water Samples

In 2010, water samples were collected in January (winter) and July (summer) from 13 water plants in Shanghai. Raw water was collected from the same sampling point for five water plants that tap on Yangtze River and likewise from the eight water plants with water supply from upstream of Huangpu River (Fig. S1). The treatment process consists of preoxidation, coagulation, sedimentation, sand filtration, and disinfection (Table 1, Fig. S2). Samples were taken at the following locations: raw water before the preoxidation; the effluent of the sedimentation basin; the effluent of the sand filtration basin; and the finished water after disinfection. Samples were collected according to US EPA (United States Environmental Protection Agency) Method 552.3/551.1 [31], [32].

**Table 1:** Summary of source water, treatment, and disinfection at 13 water plants.

Water plants	Source	Season	Treatment	Disinfectant
1–5	Yangtze River	Winter	Pre-free chlorine, coagulation, sedimentation, sand filtration	Chloramines
		Summer	Pre-free chlorine, coagulation, sedimentation, sand filtration	Chloramines
6, 8–12	Huangpu River	Winter	Pre- free chlorine, coagulation, sedimentation, sand filtration	Chloramines
		Summer	Pre- free chlorine and ammonia, coagulation, sedimentation,sand filtration	Chloramines
7	Huangpu River	Winter	Ozone, coagulation, sedimentation, sand filtration,activated carbon	Chloramines
		Summer	Ozone, coagulation, sedimentation, sand filtration,activated carbon	Chloramines
13	Huangpu River	Winter	Pre- free chlorine, coagulation, sedimentation, sand filtration	Chloramines
		Summer	Ozone, coagulation, sedimentation, sand filtration,activated carbon, ultraviolet	Chloramines

### DBP Analysis

IAA and HAA_9_ were quantified using a method similar to the US EPA Method 552.3 [31]. The HAAs were converted to their methyl esters and measured using gas chromatography/electron capture detector (GC/ECD) (Shimadzu). IF and THM_4_ were analyzed based on US EPA Method 551.1 [32]. The analytes were measured using GC/ECD. Quality assurance showed good reproducibility of the method, and limits of detection were typically in the low µg/L range (Table S1).

### Chloride, Bromide and Iodide Analysis

Chloride and bromide were quantified by ion chromatography using the US EPA recommended Method 300.1 [33], analyzed using ion chromatography (Dionex, USA). Iodide was determined using a modified Maros’s method [34]. After 0.2 mL of 0.5 g/L sodium thiosulfate, 0.1 mL of 2.5 M sulfuric acid, 0.5 mL 2-Butanone, and 1 mL of 0.5 g/L potassium dichromate were added to the water sample (10 mL), sample was shaken for 1 min, and sat for 10 min. The mixture was then extracted with 10 mL cyclohexane for 2 min. After discarding the lower phase of the mixture, the organic layer was cleaned with 5 mL water twice and dried with anhydrous Na_2_SO_4_. Extracts were analyzed with the same instrument and column as for DBP analysis. Under the splitless injection mode, 1 µl of sample was injected into the column inlet. Helium was used as the carrier gas at a flow rate of 2 ml/min (constant flow). The initial temperature was 65°C for 3 mins and increased to 80°C at a rate of 5°C/min for 3 min, finally post ran at 240°C for 5 min. The injector temperature was 230°C and the detector temperature was 260°C. Quality assurance showed good reproducibility of the method, and limits of detection were typically in the low µg/L range (Table S1).

### pH, Ammonia Nitrogen (NH_3_-N), Dissolved Organic Carbon (DOC), UV Absorbance Analysis

Water samples from the 13 water plants were also collected for pH, NH_3_-N, DOC, and UV absorbance analysis. NH_3_-N was quantified using a Nessler’s reagent spectrophotometry method [35]. The NOM content was characterized using two parameters, DOC and UV_254_. DOC was measured using TOC analyzer (Sievers). UV_254_ (for detecting humic substance) was determined using a UV/VIS Double beam spectrophotometer (Unico), and specific UV absorbance (SUVA) was calculated as (UV_254_/DOC ×100.

### Statistical Analysis

Data were analyzed using SPSS software (version 13.0, SPSS, Chicago, IL). The relationships between the variables were examined by simple Spearman correlation analysis. The nonparametric test was conducted to determine the variance of DBPs between oxidants. The statistical tests were two-tailed with significance levels of 0.05.

## Results and Discussion

### Overview of Raw Water Characterization

The characteristics of raw water are presented in Table 2 and Fig. S3. The pH values ranged from 7.0 to 7.7 in raw water among the 13 drinking water plants. The median pH value (7.5) of the Yangtze River was similar to the Huangpu River (7.2). In winter, the medians NH_3_-N concentration of the Yangtze and Huangpu Rivers were higher, compared with summer due to lower surface runoff and slower nitrification processes. Owing to organic contamination in the Huangpu River, a higher level of NH_3_-N was detected compared with the Yangtze River for the winter season (i.e., 0.98 versus 0.35 mg/L) and summer (i.e., 0.10 versus 0.06 mg/L). DOC and UV_254_ are indicators of dissolved organic compounds and humic substances, respectively. The ranges of DOC in the Yangtze and Huangpu Rivers were 2.4–4.2 mg/L and 5.8–13.3 mg/L, respectively. Similarly, higher UV_254_ levels were from the Huangpu River. This indicated that the water quality of Huangpu River was more affected by organic compounds and humic substances. However, the changes of DOC and UV_254_ levels were not the same in different seasons. DOC of the two rivers was high in summer owing to surface runoff. Conversely, in winter, the level of UV_254_ of the two rivers was not low. Seasonal variations of upriver tend to have higher humic substance level. Another possibility is the cumulative effect of organic compounds because biological activity is lower during winter [36].

**Table 2:** Raw water characteristics: comparison of water source and season.

	Yangtze River						Huangpu River					
Parameters	Winter			Summer			Winter			Summer		
	Min	Median	Max	Min	Median	Max	Min	Median	Max	Min	Median	Max
**pH**	7.2	7.4	7.6	7.4	7.6	7.7	7.0	7.2	7.4	7.1	7.4	7.6
**NH_3_-N (mg/L)**	0.33	0.35	0.39	0.01	0.06	0.40	0.72	0.98	1.56	0.05	0.10	0.21
**DOC (mg/L)**	2.4	2.9	3.9	2.4	3.8	4.2	5.8	6.2	8.2	6.3	7.1	13.3
**UV_254_ (1/cm)**	0.18	0.22	0.23	0.11	0.12	0.13	0.22	0.27	0.32	0.16	0.25	0.35
**SUVA (L/(mg·m))**	4.6	7.6	9.6	2.7	3.0	5.7	3.4	4.2	5.2	2.2	3.3	4.7
**Chloride (mg/L)**	37	39	49	11	13	14	75	82	84	57	60	66
**Bromide (µg/L)**	101	102	146	20	24	45	302	322	328	194	206	223
**Iodide (µg/L)**	6	7	7	3	4	9	12	16	18	15	16	20
**IAA (µg/L)**	0	0	0	0	0	0	0	0	0	0	0	0
**IF (µg/L)**	0	0	0	0	0	0	0	0	0	0	0	0
**HAA_9_ (µg/L)**	1.30	2.13	3.61	<1	<1	<1	4.25	4.80	5.93	<1	1.26	2.56
**THM_4_ (µg/L)**	<1	<1	<1	ND	ND	ND	<1	<1	<1	ND	3.29	9.41

Note: DOC: dissolved organic carbon. SUVA: specific UV absorbance. IAA: iodoacetic acid. IF: iodoform. HAA_9_: chloro-, bromo-, dichloro-, dibromo-, bromochloro-, bromodichloro-, dibromochloro-, trichloro-, and tribromoacetic acid. THM_4_: chloroform, bromoform, bromodichloromethane,and chlorodibromomethane. ND: not detected.

SUVA in the Yangtze River ranged from 2.7 to 9.6 L/(mg·m), and from 2.2 to 5.2 L/(mg·m) in the Huangpu River. When comparing the median levels of SUVA in raw water from the Huangpu River against those from the Yangtze River in winter, SUVA was higher in the Yangtze River (7.6 versus 4.2 L/(mg·m)). However, they were similar in summer (3.0 versus 3.3 L/(mg·m)). In contrast, the Yangtze River, with a lower DOC, had a higher SUVA in winter, suggesting that the NOM contained higher carbon aromacity and was hydrophobic in character [30].

Higher chloride, bromide, and iodide levels were expected in raw waters from the Yangtze River due to salt water intrusion. However, it is interesting to note that the concentrations of chloride, bromide, and iodide in the Huangpu River during winter were higher than that of water from the Yangtze River. For example, the median concentrations were twice as high as those from the Yangtze River in winter (i.e., 39 versus 82 mg/L, 102 versus 322 µg/L, and 7 versus 16 µg/L, respectively) (Table 2). Richardson et al. [18] also reported higher bromide and iodide levels in an inland location which had fossilized seawater. It is very likely that the Huangpu River was suffering from potential industrial and agricultural pollution, as well as contamination by natural organic compounds, such as humic substances. In winter, surface runoff decreased which led to higher concentrations of these compounds being detected in our samples.

### Occurrence of I-DBPs

Our results found that both IAA and IF were presented in finished water of 13 water plants in Shanghai, their concentrations ranged from 0.03 to 1.66 µg/L, with the highest concentration of 1.66 µg/L for IAA and 1.25 µg/L for IF, respectively (Table 3, Fig. S4). It is worth noting that IAA and IF were generally not detected in the corresponding raw water, suggesting that IAA and IF are formed during water treatment. Compared with samples collected during summer, IAA and IF were often of higher concentrations in the treated water of both rivers in winter. The levels of IAA and IF in treated water from the Huangpu River were higher than those from the Yangtze River, not only in winter (i.e., 1.08 versus 0.39 µg/L and 0.85 versus 0.18 µg/L, respectively) but also in summer (i.e., 0.27 versus 0.04 µg/L and 0.40 versus 0.01 µg/L, respectively).

**Table 3:** Finished water characteristics: comparison of water source and season.

	Yangtze River							Huangpu River					
Parameters	Winter			Summer			Winter				Summer		
	Min	Median	Max	Min	Median	Max	Min		Median	Max	Min	Median	Max
**pH**	7.1	7.3	7.4	7.5	7.7	7.9	6.7		7.1	7.2	6.9	7.2	7.6
**NH_3_-N (mg/L)**	0.20	0.24	0.35	0.17	0.32	0.35	0.57		0.77	1.30	0.21	0.42	0.85
**DOC (mg/L)**	2.1	2.6	3.1	2.4	3.2	3.7	3.3		5.3	5.7	3.9	5.4	5.9
**UV_254_ (1/cm)**	0.04	0.04	0.05	0.02	0.03	0.04	0.03		0.09	0.10	0.02	0.09	0.12
**SUVA (L/(mg·m))**	1.2	1.7	2.1	0.5	1.0	1.1	0.9		1.7	2.4	0.4	1.7	2.1
**Chloride (mg/L)**	39	45	49	15	17	21	79		87	93	64	71	75
**Bromide (µg/L)**	47	74	104	1	8	14	199		270	289	115	149	270
**Iodide (µg/L)**	2	3	7	2	5	5	<1		6.2	18	ND	5	11
**IAA (µg/L)**	0.24	0.39	0.51	0.03	0.04	0.04	0.49		1.08	1.66	0.05	0.27	0.46
**IF (µg/L)**	0.16	0.18	0.40	0.01	0.01	0.01	0.49		0.85	1.25	0.23	0.40	0.56
**HAA_9_ (µg/L)**	5.34	8.67	28.76	17.73	22.19	48.55	3.31		16.45	29.52	4.18	22.43	28.53
**THM_4_ (µg/L)**	0.60	4.09	30.51	40.12	56.91	63.74	0.60		4.47	10.34	19.83	24.50	38.33

Note: DOC: dissolved organic carbon. SUVA: specific UV absorbance. IAA: iodoacetic acid. IF: iodoform. HAA_9_: chloro-, bromo-, dichloro-, dibromo-, bromochloro-, bromodichloro-, dibromochloro-, trichloro-, and tribromoacetic acid. THM_4_: chloroform, bromoform, bromodichloromethane, and chlorodibromomethane. ND: not detected.

The concentrations of HAA_9_ ranged from 3.31 to 48.55 µg/L and THM_4_ ranged from 0.28 to 63.74 µg/L, with median values of 19.59 and 23.50 µg/L in the finished water,respectively. The levels of HAA_9_ in finished water from the Yangtze and Huangpu Rivers were similar. However, in summer, the concentration of THM_4_ in finished water from the Yangtze River (median of 56.91 µg/L) was twice as high as those from the Huangpu River (median of 24.50 µg/L). Nevertheless, all the regulated HAAs and THMs did not exceed the regulated maximum contaminant levels.

### Relationship between Water Quality/Treatment and Iodinated/Regulated DBPs Concentrations/Species Distributions

Previous studies have suggested that *p*H, DOC, UV_254_, SUVA, and iodide levels of raw water could contribute to the formation of I-DBPs [18], [37]. The Spearman correlations between raw water quality and DBPs in finished water based on this study are shown in Table 4. The parameters that positively influence IAA were NH_3_-N, pH, iodide, and UV_254;_ for IF, were UV_254_, iodide, NH_3_-N, pH, and DOC (Table 4). We found that iodide had a close relationship with chloride and bromide (Table S2), suggesting positive correlations between chloride/bromide and potential formation of IAA/IF.

**Table 4:** Simple correlation between raw water quality and DBPs in finished water.

	pH	NH_3_-N	DOC	UV_254_	SUVA	Chloride	Bromide	Iodide
**IAA**	−0.571*	0.769*	0.204	0.468*	0.221	0.781*	0.791*	0.478*
**IF**	−0.529*	0.610*	0.503*	0.699*	0.028	0.901*	0.920*	0.654*
**HAA_9_**	0.170	−0.245	−0.085	−0.245	−0.215	−0.211	−0.167	−0.060
**THM_4_**	0.423*	−0.725*	−0.076	−0.444*	−0.379	−0.607*	−0.586*	−0.362

Note: *P<0.05. IAA: iodoacetic acid. IF: iodoform. HAA_9_: chloro-, bromo-, dichloro-, dibromo-, bromochloro-, bromodichloro-, dibromochloro-, trichloro-, and tribromoacetic acid. THM_4_: chloroform, bromoform, bromodichloromethane, and chlorodibromomethane.

DOC: dissolved organic carbon. SUVA: specific UV absorbance.

The pH was considered to be an important factor in I-DBPs formation. Ye et al. [38] reported that the formation of IAA was favored under acidic conditions. The process of I-DBPs formation depends on the rate of iodide oxidation to iodate and the reaction with hypoiodous acid (HOI) and NOM [37], [39]. Further, if the raw water was low in pH, the rate of oxidation reactions to form iodite and iodate was significantly reduced. At the same time, HOI has a longer half life at low pH and, hence, more opportunity to react with NOM to form I-DBPs. Our data showed that the levels of IAA and IF were often higher in finished water from the Huangpu River than that from the Yangtze River, especially during winter. However, pH of the Huangpu River and Yangtze Rivers were similar (median of 7.2 and 7.4) in winter. Correlation analysis also demonstrated a general negative correlation between pH and IAA/IF (Table 4), although UV_254_ might be an interfering factor because of the close relationship between UV_254_ and pH (Table S2).

In general, NOM (such as humic substances) is known to be one of main influential precursors for the formation of I-DBPs, usually, the higher the NOM level, the higher the concentration of I-DBPs. Moreover, increasing iodide levels in raw water increased the formation of I-DBPs [18]. Our results demonstrated that the levels of I-DBPs were significantly higher in finished water of the Huangpu River than those of the Yangtze River (Table 3). High levels of UV_254_, and iodide in the Huangpu River contributed to the formation of I-DBPs. Correlation analyses between UV_254/_iodide levels and IAA/IF were evident (Table 4). These findings are consistent with an earlier report [18].

Almost all of the water plants used conventional treatment processes, including peroxidation (chlorine or chloramines), coagulation, sedimentation, sand filtration, and disinfection except for water plants 7 and 13 (Table 1). In summer, water plants 1 to 5 used only chlorine for preoxidation because of the low ammonia level in the source water from the Yangtze River and it also offers better water quality. On the contrary, in order to reduce regulated DBPs formation water plants 6, 8, 9, 10, 11, and 12 added ammonia to the raw water to react with chlorine for chloramination. Whereas, in winter, because of the high levels of ammonia in raw water, most of water plants used free chlorine for preoxidation, as free chlorine would react with ammonia in raw water to form chloramines.

Compared to chlorine, chloramines could reduce regulated DBPs formation [18]. However, chloramination was also known to enhance the formation of I-DBPs [18], [19], [30], [40], [41], [42]. Both chlorine and chloramines can oxidize iodide to HOI, but chlorine, not chloramines, rapidly converts HOI to iodate [37], [39]. Hence HOI has a higher chance to react with NOM to form I-DBPs during chloramination. Our results also showed that the levels of THM_4_ were low owing to the use of chloramines as disinfectant in the Yangtze River (Fig. S5). The levels of IAA and IF in water plants using chloramines were higher than those using chlorine in the Yangtze River. This is consistent with the findings of Richardson et al. [18].

Ozone and biological activated carbon were used in water plants 7 and 13. On the basis of earlier studies, pre-ozonation could provide some benefits in controlling regulated and iodinated DBPs in waters [43]–[46]. As shown in Fig. S5, Ozone could reduce the formation of HAA_9_ (median  = 4.18 µg/L) compared with chloramines (median  = 21.40 µg/L) in finished water from the Huangpu River. However, we did not observe that ozone significantly reduced I-DBPs formation when using chloramines as disinfectant in the treatment processes (Fig. S5). Furthermore, ozone has the potential to form oxygen-containing DBPs (e.g., bromate) in raw water that contain high bromide [43], [47]. In certain cases, when bromide concentrations are above 50 µg/L, it may be necessary to use other control measures to lower bromate formation (such as lowering of pH, ammonia addition) [48]. However, in the Huangpu River bromide concentrations sometimes can be higher than 50 µg/L, although the bromate level of finished water is below 10 µg/L [49]. It is believe that the high levels of NH_3_-N, and NOM in Huangpu River water might inhibit bromate formation, as suggested by an earlier study [48].

In this study we did not observe water quality parameters had a significant correlation with HAA_9_, although NOM showed negative correlations with monochloroacetic acid, dichloroacetic acid, bromochloroacetic acid, bromodichloroacetic acid, chloroform, and bromodichloromethane (Table S3). Only pH demonstrated positive correlations with monochloroacetic acid and dichloroacetic acid. Similarly, UV_254_, chloride, and bromide had negative correlation with THM_4_ (Table 4). These results are rather unexpected when compared to earlier studies [50]–[52]. The main reason for these differences is that there could be many factors affect the formation of HAAs/THMs. Further, the self-relations among these parameters are also complex (Table S2). Additionally, we also observed that HAAs/THMs concentrations in drinking water of Shanghai are low and this could again affected by many factors. We believe the current data available may not be adequate to address the relationships between HAAs/THMs and water quality parameters. More water samples with higher sampling frequency are necessary to reveal the relationships between various parameters to that of the regulated and unregulated DBPs distributions in drinking water in the future study.

IAA and IF commonly occurred in high iodide and chloraminated drinking waters, but the levels of IAA and IF were relatively low. The maximum concentrations of IAA and IF were 1.66 µg/L and 1.25 µg/L, respectively, and most of the time they were at sub-ppb or ppt levels. Nevertheless, it is important to know whether IAA and IF could have potential adverse human health risks, at these levels in drinking water. It is suggested that both IAA and IF in drinking water should be monitored routinely since it is an issue of public health concern.

### Conclusions

The levels of IAA and IF in finished water from the Huangpu River were generally higher than those from the Yangtze River as the water quality in the Huangpu River has been noted to be deteriorated over the past years. Low *p*H, increasing iodide, NH_3_-N, and NOM levels in the raw waters further increased the formation of IAA and IF. Natural iodide presented in the water sources could lead to higher concentrations of I-DBPs in drinking water. We also noted that chloramines were more effective in reducing the levels of THMs compared with chlorine. Organic matter pollutants led to higher halide ion in the Huangpu River than that caused by salinity intrusion in the Yangtze River. In short, this study provides useful information concerning the presence of various regulated and newly emerging DBPs in the water plants in Shanghai. The findings also offer scientific basis for a better understanding on the formation of IAA and IF in the drinking water. We trust these data are useful not only for the water suppliers and regulators in China but also other parts of the world.

## Supporting Information

Figure S1 Distribution of 13 water plants in Shanghai. ▴ denotes water plants with the Yangtze River as the raw water and • denotes water plants with the Huangpu River as the raw water.
(TIF)

Figure S2 The treatment processes of 13 drinking water plants.
(TIF)

Figure S3 Raw water characteristics: Comparison of the Yangtze River and the Huangpu River in winter and summer. (A) Distribution of pH values in different rivers and seasons. (B) Distribution of NH_3_-N and DOC values in different rivers and seasons. (C) Distribution of UV_254_ and SUVA values in different rivers and seasons. (D) Distribution of chloride, bromide, and iodide in different rivers and seasons. (E) Distribution of DBPs in different rivers and seasons.
(TIF)

Figure S4 Finished water characteristics: Comparison of the Yangtze River and the Huangpu River in winter and summer. (A) Distribution of pH values in different rivers and seasons. (B) Distribution of NH_3_-N and DOC values in different rivers and seasons. (C) Distribution of UV_254_ and SUVA values in different rivers and season. (D) Distribution of chloride, bromide, and iodide in different rivers and seasons. (E) Distribution of DBPs in different rivers and seasons.
(TIF)

Figure S5 Relationship between oxidants and DBPs formation in finished water, showing the comparison between water plants using chloramines, ozone and chlorine in the Yangtze River and the Huangpu River. *P < 0.05 vs. chlorine, # *P* < 0.05 vs. ozone.
(TIF)

Table S1 The parameters of detected methods for IAA, IF, THM_4_, HAA_9_, and halide ion.
(DOCX)

Table S2 Simple correlation among raw water quality.
(DOCX)

Table S3 Simple correlation between raw water quality and single HAA/THM.
(DOCX)
