# Supplementary figures and images for: Occurrence of Regulated and Emerging Iodinated DBPs in the Shanghai Drinking Water
Source: PLoS One. 2013 Mar 26;8(3):e59677. doi: 10.1371/journal.pone.0059677 (PMC3608560; doi:10.1371/journal.pone.0059677)

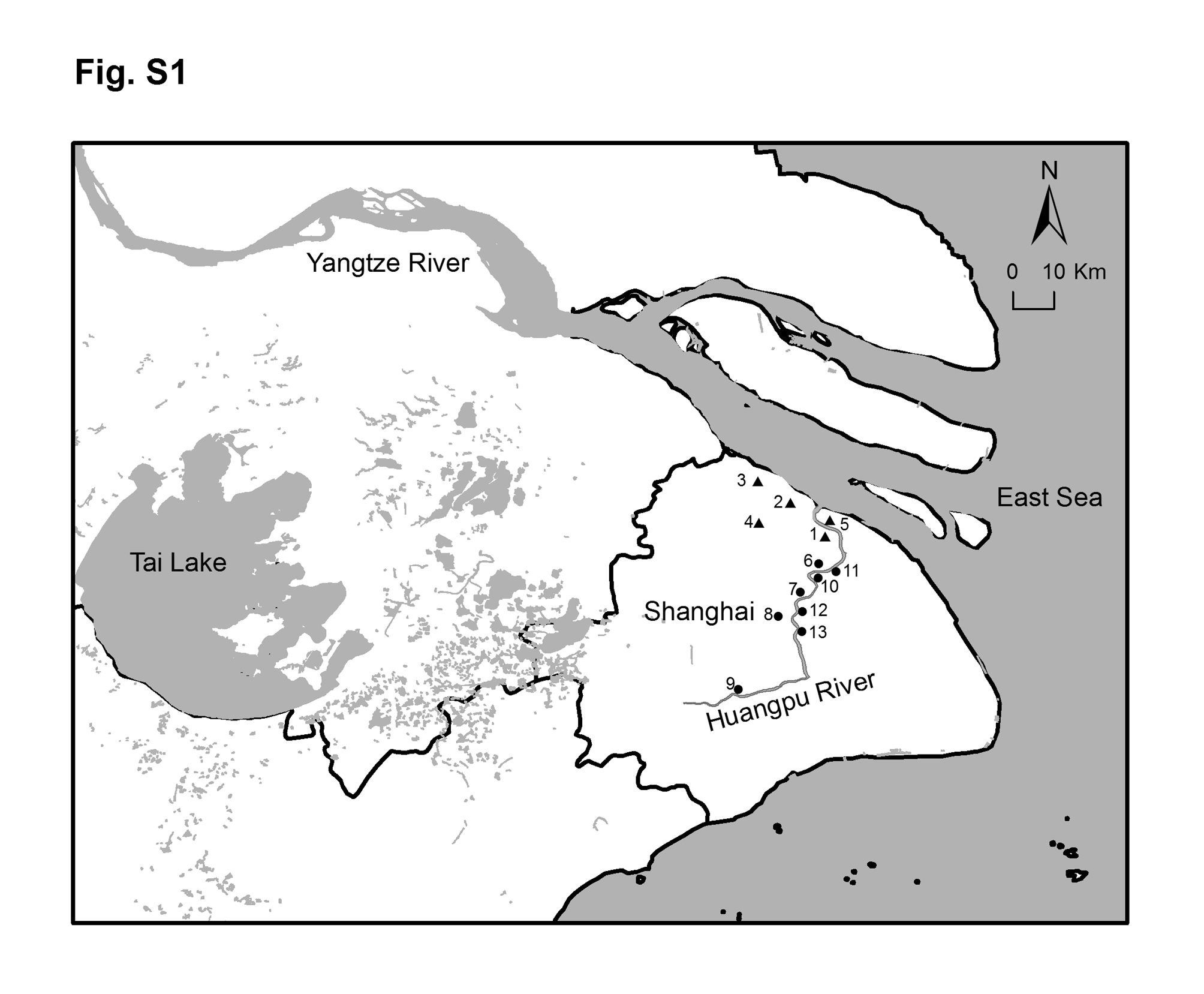

Supplement: Figure S1 — Distribution of 13 water plants in Shanghai. ▴ denotes water plants with the Yangtze River as the raw water and • denotes water plants with the Huangpu River as the raw water. (TIF) [file pone.0059677.s001.tif]

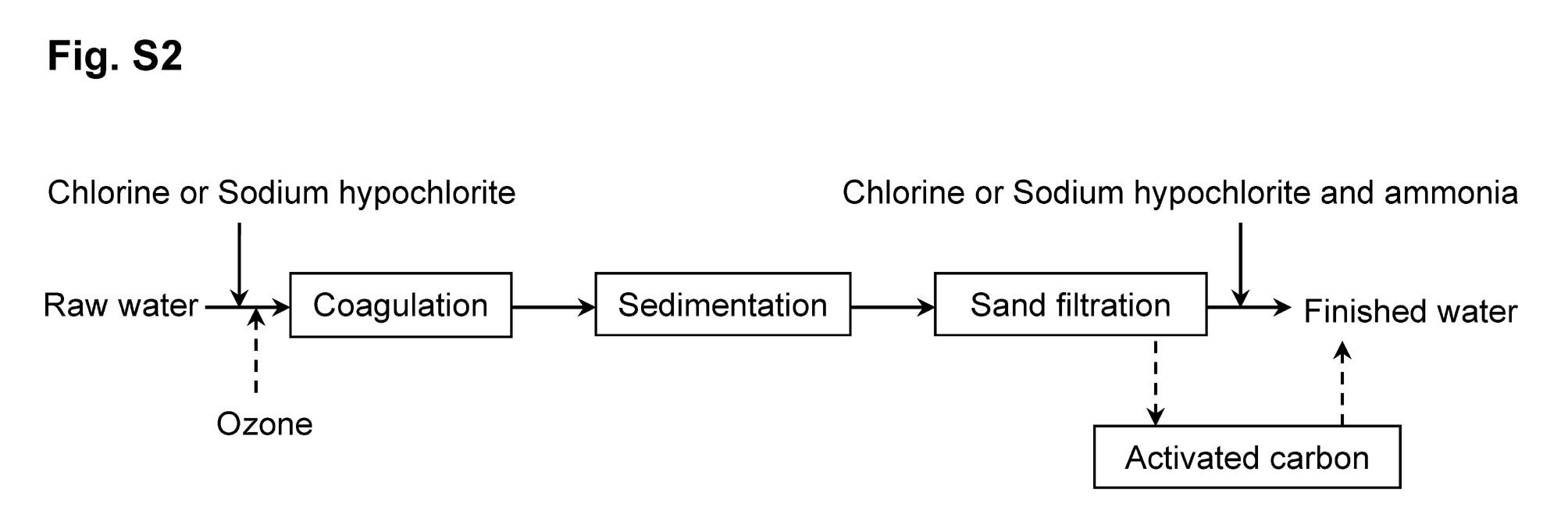

Supplement: Figure S2 — The treatment processes of 13 drinking water plants. (TIF) [file pone.0059677.s002.tif]

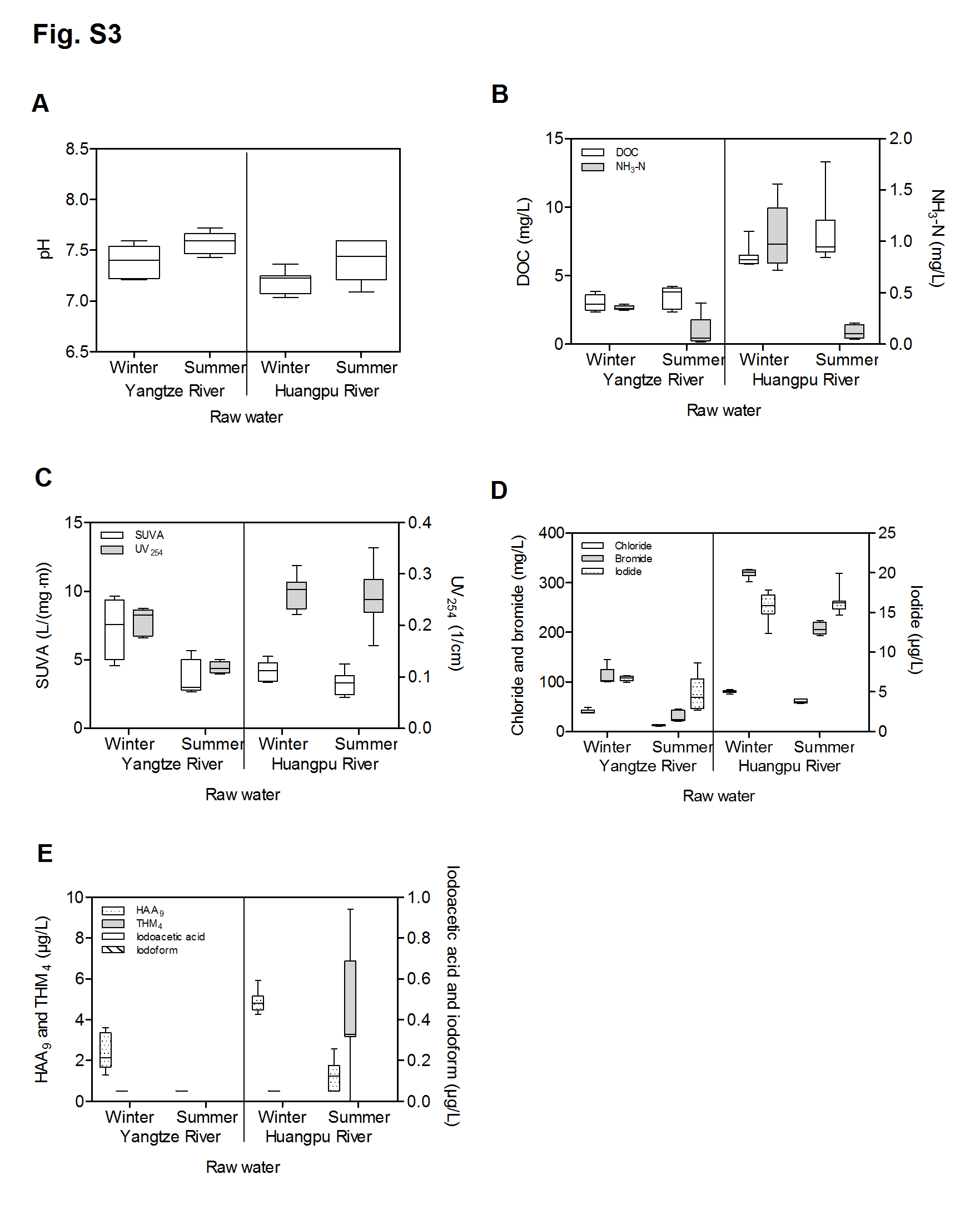

Supplement: Figure S3 — Raw water characteristics: Comparison of the Yangtze River and the Huangpu River in winter and summer. (A) Distribution of pH values in different rivers and seasons. (B) Distribution of NH3-N and DOC values in different rivers and seasons. (C) Distribution of UV254 and SUVA values in different rivers and seasons. (D) Distribution of chloride, bromide, and iodide in different rivers and seasons. (E) Distribution of DBPs in different rivers and seasons. (TIF) [file pone.0059677.s003.tif]

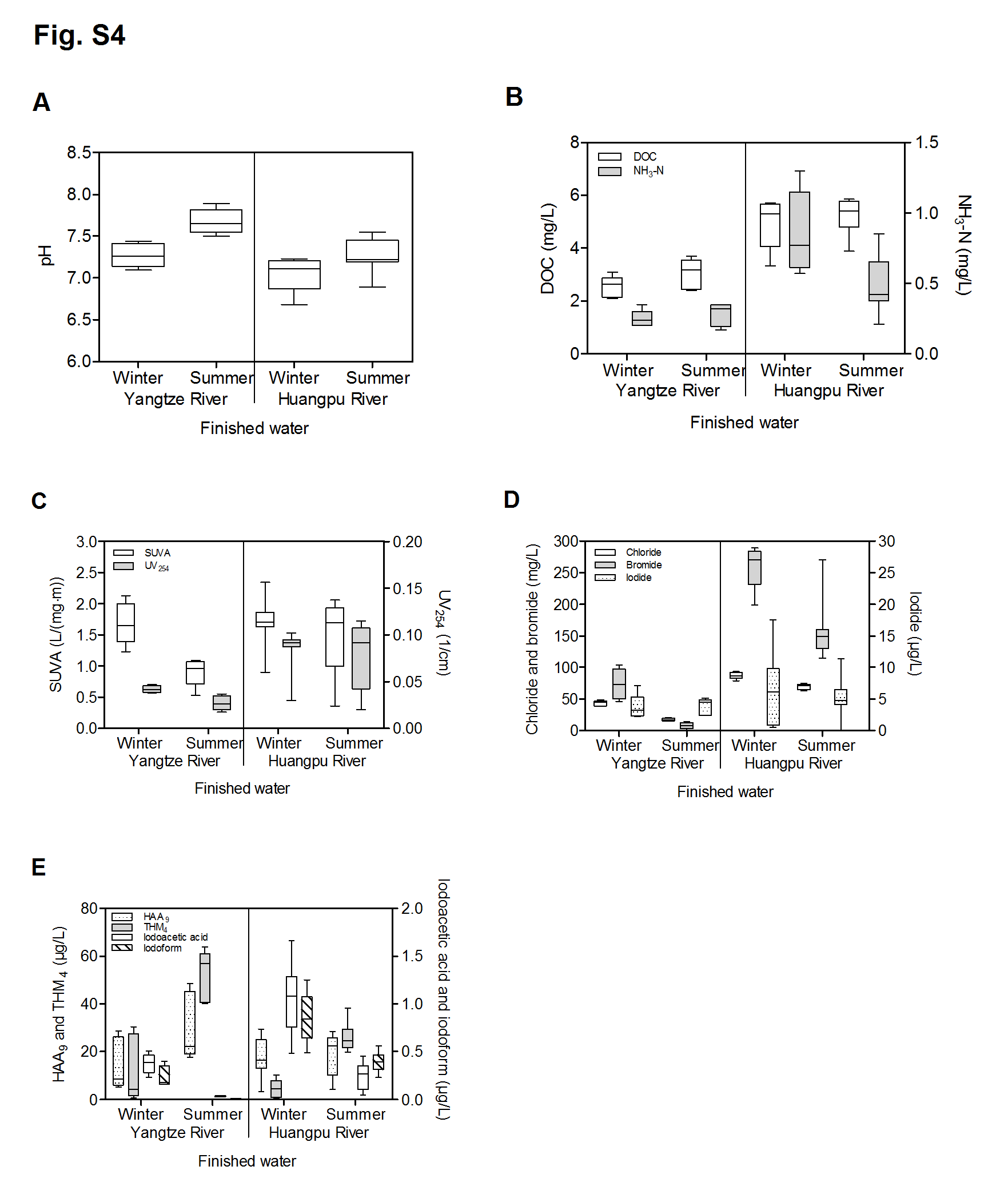

Supplement: Figure S4 — Finished water characteristics: Comparison of the Yangtze River and the Huangpu River in winter and summer. (A) Distribution of pH values in different rivers and seasons. (B) Distribution of NH3-N and DOC values in different rivers and seasons. (C) Distribution of UV254 and SUVA values in different rivers and season. (D) Distribution of chloride, bromide, and iodide in different rivers and seasons. (E) Distribution of DBPs in different rivers and seasons. (TIF) [file pone.0059677.s004.tif]

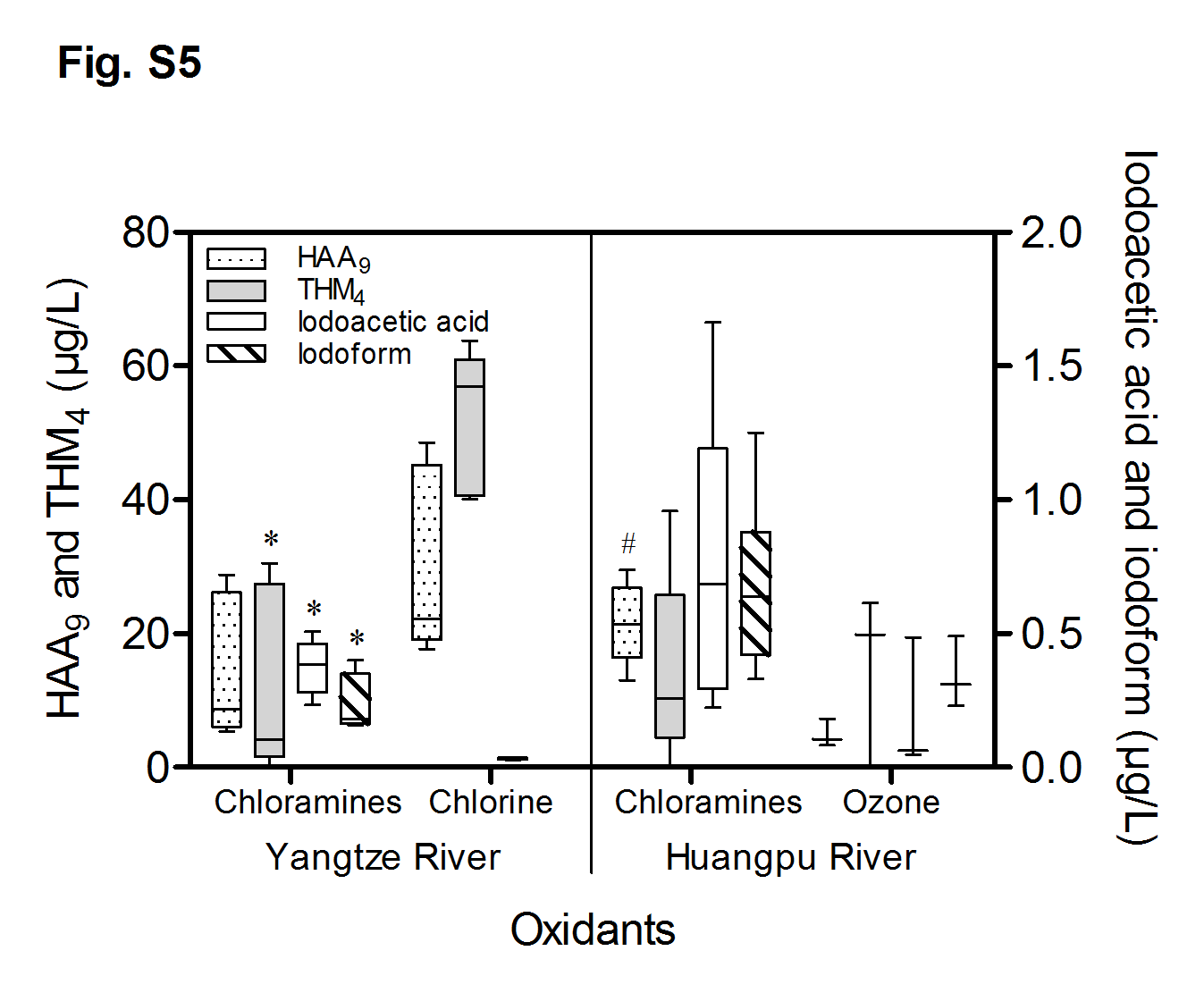

Supplement: Figure S5 — Relationship between oxidants and DBPs formation in finished water, showing the comparison between water plants using chloramines, ozone and chlorine in the Yangtze River and the Huangpu River. *P < 0.05 vs. chlorine, # P < 0.05 vs. ozone. (TIF) [file pone.0059677.s005.tif]
