# Supplementary material for: Occurrence of Regulated and Emerging Iodinated DBPs in the Shanghai Drinking Water
Source: PLoS One. 2013 Mar 26;8(3):e59677. doi: 10.1371/journal.pone.0059677 (PMC3608560; doi:10.1371/journal.pone.0059677)
Supplement: Table S1 — The parameters of detected methods for IAA, IF, THM4, HAA9, and halide ion. (DOCX) [file pone.0059677.s006.docx]

**Table S1.** The parameters of detected methods for IAA, IF, THM_4_, HAA_9_, and halide ion.

| **Compound** | **Linear range (μg/L)** | **r^2^** | **MDL (μg/L)** | **Concentration (μg/L)** | **Recovery (%)** | **RSD (%) (n=7)** |
| --- | --- | --- | --- | --- | --- | --- |
| IAA | 0.01-0.2 | 0.994 | 0.004 | 0.05 | 98 | 7.0 |
|  | 0.05-2.5 | 0.999 | 0.004 | 0.5 | 111 | 2.1 |
| IF | 0.05-5 | 0.999 | 0.018 | 0.5 | 97 | 3.5 |
| CF | 1-100 | 0.999 | 0.327 | 10 | 105 | 2.3 |
| BDCM | 1-100 | 0.998 | 0.315 | 10 | 110 | 2.4 |
| CDBM | 1-100 | 0.999 | 0.345 | 10 | 107 | 2.3 |
| BF | 1-100 | 0.998 | 0.207 | 10 | 101 | 2.7 |
| CAA | 1-100 | 0.999 | 0.502 | 10 | 102 | 2.9 |
| BAA | 1-100 | 0.999 | 0.306 | 10 | 104 | 1.8 |
| DCAA | 1-100 | 0.999 | 0.272 | 10 | 105 | 1.7 |
| TCAA | 1-100 | 0.999 | 0.296 | 10 | 108 | 1.7 |
| BCAA | 1-100 | 0.999 | 0.280 | 10 | 104 | 1.8 |
| DBAA | 1-100 | 0.999 | 0.318 | 10 | 111 | 2.2 |
| BDCAA | 1-100 | 0.999 | 0.335 | 10 | 97 | 3.3 |
| CDBAA | 1-100 | 0.999 | 0.387 | 10 | 95 | 4.1 |
| TBAA | 1-100 | 0.998 | 0.191 | 10 | 94 | 4.0 |
| Chloride | 1000-50000 | 0.999 | 55.600 | 10000 | 90 | 1.3 |
|  | 50000-1000000 | 0.999 | 55.600 | 250000 | 99 | 0.4 |
| Bromide | 1-50 | 0.999 | 0.370 | 25 | 99 | 2.9 |
|  | 50-1000 | 0.999 | 0.370 | 250 | 112 | 0.7 |
| Iodide | 1-100 | 0.999 | 0.027 | 10 | 102 | 5.1 |

Note: MDL: method detection limit. RSD: relative standard deviation. IAA: iodoacetic acid. IF: iodoform. CF: chloroform. BDCM: bromodichloromethane. CDBM: dibromochloromethane. BF: bromoform. CAA: monochloroacetic acid. BAA: monobromoacetic acid. DCAA: dichloroacetic acid. TCAA: trichloroacetic acid. BCAA: bromochloroacetic acid. DBAA: dibromoacetic acid. BDCAA: bromodichloroacetic acid. CDBAA: chlorodibromoacetic acid. TBAA: tribromoacetic acid
