# Supplementary material for: Occurrence of Regulated and Emerging Iodinated DBPs in the Shanghai Drinking Water
Source: PLoS One. 2013 Mar 26;8(3):e59677. doi: 10.1371/journal.pone.0059677 (PMC3608560; doi:10.1371/journal.pone.0059677)
Supplement: Table S2 — Simple correlation among raw water quality. (DOCX) [file pone.0059677.s007.docx]

**Table S2.** Simple correlation among raw water quality.

|  | pH | NH_3_-N | DOC | UV_254_ | SUVA | Chloride | Bromide | Iodide |
| --- | --- | --- | --- | --- | --- | --- | --- | --- |
| pH | 1.000 |  |  |  |  |  |  |  |
| NH_3_-N | -0.552* | 1.000 |  |  |  |  |  |  |
| DOC | -0.321 | -0.020 | 1.000 |  |  |  |  |  |
| UV_254_ | -0.552* | 0.304 | 0.586* | 1.000 |  |  |  |  |
| SUVA | -0.113 | 0.154 | -0.620* | 0.153 | 1.000 |  |  |  |
| Chloride | -0.615* | 0.645* | 0.625* | 0.716* | -0.132 | 1.000 |  |  |
| Bromide | -0.607* | 0.614* | 0.624* | 0.770* | -0.091 | 0.981* | 1.000 |  |
| Iodide | -0.255 | 0.360 | 0.692* | 0.490* | -0.391* | 0.754* | 0.710* | 1.000 |

Note: **P* ＜ 0.05. DOC: dissolved organic carbon. SUVA: specific UV absorbance
