# Supplementary material for: Occurrence of Regulated and Emerging Iodinated DBPs in the Shanghai Drinking Water
Source: PLoS One. 2013 Mar 26;8(3):e59677. doi: 10.1371/journal.pone.0059677 (PMC3608560; doi:10.1371/journal.pone.0059677)
Supplement: Table S3 — Simple correlation between raw water quality and single HAA/THM. (DOCX) [file pone.0059677.s008.docx]

**Table S3.** Simple correlation between raw water quality and single HAA/THM.

|  | pH | NH_3_-N | DOC | UV_254_ | SUVA | Chloride | Bromide |
| --- | --- | --- | --- | --- | --- | --- | --- |
| CF | 0.493* | -0.694* | -0.241 | -0.504* | -0.214 | -0.667* | -0.634* |
| BDCM | 0.459* | -0.704* | -0.123 | -0.460* | -0.338 | -0.613* | -0.599* |
| CDBM | 0.280 | -0.679* | 0.240 | -0.204 | -0.526* | -0.372 | -0.357 |
| BF | 0.208 | -0.689* | 0.516* | 0.046 | -0.587* | -0.219 | -0.202 |
| CAA | 0.459* | -0.595* | 0.071 | -0.420* | -0.448* | -0.370 | -0.375 |
| BAA | 0.000 | 0.000 | 0.000 | 0.000 | 0.000 | 0.000 | 0.000 |
| DCAA | 0.456* | -0.542* | -0.026 | -0.406* | -0.348 | -0.371 | -0.351 |
| TCAA | -0.092 | -0.101 | 0.453* | 0.191 | -0.381 | 0.281 | 0.311 |
| BCAA | 0.209 | -0.083 | -0.407 | -0.497* | -0.088 | -0.302 | -0.284 |
| DBAA | -0.270 | -0.055 | 0.536* | 0.368 | -0.290 | 0.366 | 0.393* |
| BDCAA | 0.145 | -0.169 | -0.624* | -0.440* | 0.186 | -0.526* | -0.494* |
| CDBAA | -0.303 | 0.215 | -0.340 | 0.149 | 0.467* | 0.034 | 0.085 |
| TBAA | -0.433* | 0.684* | -0.366 | 0.222 | 0.623* | 0.349 | 0.356 |

Note: **P* ＜ 0.05. CF: chloroform. BDCM: bromodichloromethane. CDBM: dibromochloromethane. BF: bromoform. CAA: monochloroacetic acid. BAA: monobromoacetic acid. DCAA: dichloroacetic acid. TCAA: trichloroacetic acid. BCAA: bromochloroacetic acid. DBAA: dibromoacetic acid. BDCAA: bromodichloroacetic acid. CDBAA: chlorodibromoacetic acid. TBAA: tribromoacetic acid. DOC: dissolved organic carbon. SUVA: specific UV absorbance.
